# Elevated Systemic Antibodies towards Commensal Gut Microbiota in Autoinflammatory Condition

**DOI:** 10.1371/journal.pone.0003172

**Published:** 2008-09-09

**Authors:** Gayane P. Manukyan, Karine A. Ghazaryan, Zhanna A. Ktsoyan, Zaruhi A. Khachatryan, Karine A. Arakelova, Denise Kelly, George Grant, Rustam I. Aminov

**Affiliations:** 1 Institute of Molecular Biology, National Academy of Sciences, Yerevan, Armenia; 2 Rowett Institute of Nutrition and Health, University of Aberdeen, Aberdeen, United Kingdom; Columbia University, United States of America

## Abstract

**Background:**

Familial Mediterranean fever (FMF) is an autoinflammatory condition, which is characterized by acute, self-limiting episodes of fever and serositis and chronic subclinical inflammation in remission. Here we investigated the consequence of this condition on the level of systemic antibodies directed towards common intestinal bacteria.

**Methodology/Principal Findings:**

The level of systemic antibodies towards the antigens of *Bacteroides*, *Parabacteroides*, *Escherichia*, *Enteroccocus* and *Lactobaccilus* was measured by ELISA in FMF patients at various stages of the disease and in healthy controls. The difference between remission and attack was not significant. IgG antibodies against the antigens of *Bacteroides*, *Parabacteroides*, *Escherichia* and *Enteroccocus* were significantly increased in FMF compared to control while IgA levels were not significantly affected. Western blot analyses demonstrated the IgG reactivity against multiple antigens of commensal bacteria in FMF. Serological expression cloning was performed to identify these antigens. No single dominant antigen was identified; the response was generalized and directed against a variety of proteins from *Bacteroides*, *Parabacteroides*, *Escherichia*, and other gut commensals.

**Conclusions/Significance:**

This autoinflammatory syndrome is characterized by the increased systemic reactivity against commensal gut microbiota. This is probably the consequence of hypersensitivity of the inflammasome in FMF that triggers the inflammation and contributes to the excessive translocation of bacteria and bacterial antigens through the gut barrier.

## Introduction

Familial Mediterranean fever [FMF, MIM249100] is one the most common hereditary autoinflammatory syndromes [1]. It occurs in populations originating from the Mediterranean basin, mainly in Jews, Armenians, Arabs, and Turks. The genetic cause of FMF is the mutated version of pyrin/marenostrin, which is primarily expressed in neutrophils, eosinophils, monocytes, dendritic cells, and sinovial fibroblasts [2], [3], [4], [5]. Clinically, the disease is characterized by acute self-resolving attacks of fever with serositis such as peritonitis, pleuritis, and arthritis, with a massive influx of polymorphonuclear leukocytes into the affected tissues. In remission the patients are clinically asymptomatic, although a number of inflammation markers are elevated suggesting persisting subclinical inflammation [6]. Antimitotic drug colchicine reduces the frequency, duration and intensity of attacks and extends the remission periods. It is also effective in prevention of FMF complications such as amyloidosis and renal failure.

Although the genetic basis of FMF is clearly defined and genotyping for the corresponding mutations becomes a standard diagnostic tool for this disease, the environmental factors such as diet, stress, or physical activity were also implicated in the initiation of disease attacks [7], [8]. In this regard, the gut mucosal interface can be considered as one of the major areas where the organism interacts with the environment, in particular, with the diverse gut microbiota. Under the normal circumstances, the passive protection of the host against commensal bacteria in the intestine is achieved by the secretion of mucin by goblet cells while the active control involves a number of nonspecific cytotoxic cells and phagocytes as well as humoral mechanisms with the synthesis and translocation of antimicrobials such as defensins [9], the production of large quantities of intestinal IgA that are secreted into the lumen [10], complement fragments, cytokines, and chemokines. The interplay between the host and gut microbiota includes sensing bacterial signals through an impressive host array of the innate immunity receptors such as TLR, NLR, and C-type lectins. This signaling appeared to be important not only for the recognition of, and launching defenses against, the bacteria but also for the normal development of immune system [11], maintenance of intestinal epithelial homeostasis, protection against gut injury and associated mortality [12], regulation of mucosal inflammation and maintenance of intestinal epithelial barrier integrity [13], [14].

The breakdown in recognition of commensal bacteria by the host may result in severe inflammatory disease. In some Crohn's disease (CD) patients, for example, mutations in a microbial product sensor NLR, the NOD2/CARD15 protein, is associated with the disease susceptibility [15], [16]. Bacterial ligand for this receptor was identified as bacterial muramyl dipeptide [17], [18] and the failure to recognize this ligand in CD patients apparently initiates the cascade(s) eventually resulting in breach of tolerance and the launch of an aggressive Th1-skewed response against the components of normal diet and commensal bacteria [19], [20], [21], [22].

Structurally, pyrin is a 781-residue protein and consists of a 92-amino acid N-terminal PYRIN (PYD) domain, a B-box zinc finger, a coiled-coil region and a ∼200-amino acid C-terminal B30.2/rfp/SPRY domain [23], with no LRR that is usually present in innate immunity receptors. The innate immunity regulation functions of pyrin are carried out through the interaction of two domains of the protein, PYRIN and B30.2/rfp/SPRY, with the proinflammatory caspase-1 activating complex, the inflammasome. The PYRIN domain binds and competes for ASC (apoptosis-associated speck-like protein containing a caspase-recruitment domain), an inflammasome component, thus decreasing its availability for the cryopyrin/NALP3/CIAS1 inflammasome. This decreases caspase-1 activation and pro-interleukin-1β processing and secretion thus lessening the extent of the inflammatory response [24]. The mutational hotspot in FMF, however, is located in the C-terminal B30.2/rfp/SPRY domain, which modulates the inflammatory response through the interaction with several components of the inflammasome. In addition to interaction with cryopyrin/NALP3/CIAS1, it interacts directly with caspase-1 and its substrate pro-IL-1β [25], [26]. The net effect of these interactions is the suppression of IL-1β activation and block of inflammation. It was suggested that the heightened IL-1β responsiveness could be a factor selecting for mutations in the *MEFV* gene [25]. Taking into consideration the overall role of pyrin as a modulator/suppressor of the inflammatory response, another factor contributing to the autoinflammatory nature of the disease could be the reduced *MEFV* messenger RNA expression in FMF [27]. First, it may contribute to the lower titer of pyrin and its PYRIN domain molecules in the cell thus making more ASC molecules available to initiate caspase-1 activation. Second, the reduced concentration of pyrin and therefore of its B30.2/rfp/SPRY domain, which, in addition, is mutated to the loss of caspase-1 suppressor function in most of FMF cases, may provoke easier triggering the inflammation cascade through caspase-1 activation. Thus, both consequences of *MEFV* mutations may lead to the heightened responsiveness of cryopyrin/NALP3/CIAS1, which can be oligomerized and activated in response to a very diverse range of ligands such as bacterial muramyl dipeptide, ATP, toxins, bacterial and viral RNA, small antiviral compounds, *Staphylococcus*, *Listeria*, and uric acid crystals as well as by low intracellular potassium concentration [28]–[33]. While these exo- and endogenous stimuli are not perceived as danger signals or are efficiently suppressed by the normal innate immunity, the facilitated oligomerization of the cryopyrin/NALP3/CIAS1, which is not adequately suppressed by the mutated pyrin in FMF, may be the underlying cause in this disease, resulting in excessive pro-caspase-1 and pro-IL1β processing. Indeed, monocytes from FMF patients in remission fail to induce LPS homologous tolerance and exhibit heightened sensitivity to bacterial endotoxin [34], one of the important activators of the cryopyrin/NALP3/CIAS1 inflammasome [35].

We hypothesized therefore that the neutrophils, monocytes and dendritic cells in the lamina propria of FMF patients may exhibit heightened sensitivity to the physiologically normal concentrations of bacterial ligands entering from the gut lumen and may provoke the episodes of acute inflammation as well as contribute to the chronic subclinical inflammation in remission. One of the consequences of such continuous stimulation of the innate immunity resulting in inflammation may be the priming of adaptive immune responses directed towards the antigens of gut commensal bacteria. To test this, we examined the systemic immune reactivity directed against gut commensal bacteria in the sera of FMF patients and healthy control subjects. We found a substantially increased IgG reactivity against multiple antigens of common gut bacteria in FMF. Using the serological expression cloning approach we also identified bacterial antigens that are involved in these immune reactions. The antigens were from the variety of gut commensals, mostly from *Bacteroides* and *Parabacteroides* species.

## Materials and Methods

### Subjects and sampling

Thirteen FMF patients (aged from 14 to 50 years old, mean age – 24.3 years) and 11 healthy volunteers (aged from 24 to 52 years old, mean age – 32.4 years) were enrolled in this study (Table 1). Blood serum and fecal samples from FMF patients were obtained from the Department of Gastroenterology and FMF at the Medical Centre *Armenia* in Yerevan, Armenia. The clinical diagnosis of FMF was based on Tel-Hashomer criteria [36] and genetic confirmation of the *MEFV* mutation carrier status was performed at the Centre of Medical Genetics in Yerevan, Armenia. Control group consisted of healthy volunteers without the family history of FMF. Except colchicine, none of the study subjects were taking any other medication at least three months prior sampling. All participants were informed about the study aims and gave their consent to participate in it. The corresponding protocols were approved by the local ethical committee at the Institute of Molecular Biology (IMB).

**Table 1:** Subjects and analytical methods applied.

Subjects	Gender	Colchicine therapy	Analysis applied
FMF87 (R)*	F	−	SE†; E††; W†††
FMF107 (R)	M	−	SE; E; W
FMF155 (R)	M	−	SE; E; W
FMF156 (R)	F	+	SE; E; W
FMF176 (R)	M	+	E
FMF177 (R)	M	+	E; W
FMF179 (R)	M	+	E; W
FMF181 (R)	M	+	E; W
FMF183 (R)	M	+	E
FMF172 (A) **	M	−	E
FMF186 (A)	M	+	E; W
FMF187 (A)	F	+	E
FMF190 (A)	M	+	E; W
C4***	F		SE; E; W
C6	F		E
Cm13	M		SE; E
C15	F		E; W
C104	M		E
C105	F		E
C106	F		E
C108	F		E; W
C109	M		E
C110	M		E; W
C111	M		E; W

* – FMF patients in remission period.

** – FMF patients in attack period.

*** – Healthy controls.

† – Serological expression cloning analysis.

†† – ELISA.

††† – Western blot analysis.

Blood samples were collected from all FMF patients and healthy subjects, and fecal samples from 6 FMF patients and 3 healthy controls (Table 1). The blood samples were centrifuged and cell-free supernatants were stored at −25°C until analyzed.

### Determination of systemic immune responses against gut bacteria

#### Isolation and identification of gut bacteria

Fresh fecal samples were collected from three FMF patients in remission period (FMF155 (R), FMF177 (R), and FMF179 (R)) and one healthy control (C 15) (Table 1). Fecal samples were placed in sterile bottles and processed within one hour after collection. Approximately 0.9 g of a fecal sample was serially diluted in 0.9% NaCl and 100 µl aliquots were spread on plates with selective and nonselective media: Wilkins-Chalgren agar, Schaedler agar, Bacteroides-Bile-Esculin agar, Blaurock agar, Reinforced-Clostridial agar, MRS agar, Endo agar, and Sabouraud Maltose agar. All anaerobic strains on the anaerobic selective media were incubated in an anaerobic chamber with a 10% CO_2_ and 90% N_2_ mix at 37°C. Plates with media for aerobic strains were incubated aerobically for 24–48 h at 37°C. A total of 120 isolates were obtained, the identity of bacterial strains obtained after purification was verified by Gram staining, microscopy, and sequencing of the 16S rRNA genes.

Colony PCR was applied to amplify the 16S rRNA gene directly from bacterial colonies (one-quarter of a one-mm colony) using the set of bacterial primers 27F (5′-AGAGTTTGATCCTGGCTCAG -3′, positions 8 to 27 in the *Escherichia coli* 16S rRNA gene) and 1492R (5′-ACGGCTACCTTGTTACGACTT-3′; positions 1510 to 1492 in the *E. coli* gene) [37]. The GoTaq PCR kit (Promega, UK) was used for amplification, with 10.0 pmol of each primer. PCR was performed as follows: one cycle of 95°C for 7 min, followed by 30 cycles of denaturation at 95°C for 30 sec, annealing at 57°C for 30 sec and elongation at 72°C for 2 min, with a final extension at 72°C for 10 min. The resulting amplicons were purified using a Wizard SV Gel and PCR Clean-Up System (Promega, UK), according to the manufacturer's instructions. PCR products were analyzed by electrophoresis on a 1% agarose gel. Sequencing primers used were: 519F (5′-CAGCAGCCGCGGTAATAC-3′, *E. coli* positions 519 to 536), 519R (5′-GTATTACCGCGGCTGCTG-3′, positions 536 to 519), 926F (5′-AAACTCAAAGGAATTGACGG-3′, positions 907 to 926), 926R (5′-CCGTCAATTCCTTTGAGTTT-3′; positions 926 to 907) [38], and the previously described 27F and 1492R. Sequences were read on an automated Beckman sequencer (Beckman, UK) and assembled with the ChromasPro v1.33 program. The almost complete 16S rRNA gene sequences were searched against the GenBank entries using on-line BLAST (http://www.ncbi.nlm.nih.gov/blast). Sequences with more than 99% similarity to the validly described taxa were considered as the same species/phylotypes.

#### Measurement of bacterial specific IgG and IgA antibodies in serum

The level of antibodies in the serum that react with bacteria was determined by a sandwich enzyme-linked immunosorbent assay (ELISA). Briefly, to prepare the bacterial lysates for ELISA the overnight colonies were scraped off from the surface of agar media and resuspended in 0.5 ml of cold carbonate-bicarbonate buffer (pH 9.6). The bacterial suspension was homogenized by a mechanical attrition procedure using Lysing Matrix E (MP Biomedicals, UK) in a mini-bead beater (FastPrep FP120) for 40 seconds. The debris was removed by 5 min centrifugation at 14,000×g and the supernatant was used as the coating antigen in ELISA assay. To ensure the reproducibility of ELISA, the protein concentration in bacterial lysates was determined using a standard Bicinchoninic acid (BCA) protein assay (Pierce, UK) and adjusted to 0.5 µg/µl.

For the assay, the cell-free bacterial extracts (0.5 µg of total protein per well) were subsequently coated onto a 96-well microtiter plate (Immulon 4 HBX, flat bottom) and kept at 4°C overnight. The plate then was washed with PBS containing 0.05% Tween 20 (PBST), and then blocked with 1% BSA in PBS for 1 h at room temperature. The blood serum samples were serially diluted in PBS (1∶500, 1∶1000, 1∶2000, and 1∶4000) and loaded onto the plate. All four dilutions against a single bacterial lysate were run on the same plate to minimize plate-to-plate variations. After incubation for 2 h at room temperature, the plate was washed and peroxidase-conjugated goat anti-human IgG (1∶12000) antibody (Sigma-Aldrich, UK) in PBS was added to each well. The plate was then left for 1 h at room temperature, washed, and the tetramethyl-benzidine (TMB) liquid substrate for ELISA (Sigma-Aldrich, UK) was added. Color development was allowed for 20 min in the dark and then stopped. The absorbance in each well was measured at 450 and 630 nm using a plate reader. The OD data were imported into the Excel software and the serum titers towards a given bacterial lysate were calculated based on an arbitrary ELISA OD cut-off value that targeted the linear areas of OD vs. dilution curves.

The evaluation of specific IgA was performed using sandwich ELISA similarly to IgG determination until the secondary antibody addition step. At this point, the goat anti-human IgA (1∶2000) antibody (Sigma-Aldrich, UK) in PBS was added and incubated for 1 h, again washed and anti-goat IgG (1∶1000) biotin conjugated antibody (Sigma-Aldrich, UK) was loaded for 1 h. Plate was washed before the addition of extravidin-peroxidase (Sigma-Aldrich, UK) diluted 1∶1000 in PBS and incubated for a further 1 h at room temperature. The addition of substrate, OD measurement, and titer calculation were performed as described above.

#### Measurement of total Ig classes (IgG, IgA, and IgM) and total IgG in serum

The evaluation of total antibody classes (IgG, IgA, and IgM) was performed by an indirect ELISA. Serum samples were serially diluted (1∶5000, 1∶10000, 1∶20000, 1∶40000, 1∶800000, 1∶160000, 1∶320000 and 1∶640000 dilution), 100 µl of each dilution was applied per well of a 96-well microtiter plate (Immulon 4HBX, flat bottom) and kept overnight at 4°C. Plate was washed with PBST and blocked with 1% BSA in PBS for 1 h at room temperature. The plate was washed and peroxidase-conjugated goat anti-human polyvalent immunoglobulins (IgG, IgA, and IgM) (1∶10000) or peroxidase-conjugated goat anti-human IgG (1∶12000) antibody (Sigma-Aldrich, UK) in PBS was added to each well for 1 h. The addition of substrate, OD measurement, and titer determination were performed as described above.

#### Immunoblotting

SDS-PAGE separation of bacterial proteins was performed by using 9% acrylamide minigels under denaturing conditions. Cell-free bacterial extracts were dissolved in 2× Laemmli sample buffer (Sigma-Aldrich, UK) and heated for 10 min at 100°C. Samples were run along with the biotinylated SDS-6B molecular weight standards (Sigma-Aldrich, UK).

Proteins were transferred to a polyvinylidine fluoride membrane (Immobilon-P, Millipore, UK) in a tank system. The membranes were blocked in PBS containing 1% BSA for 1 h, washed with PBS, and incubated with the human serum diluted to 1∶3000 in PBS with 1% BSA at 4°C overnight. After the wash with PBS, the membranes were incubated with peroxidase-conjugated goat anti-human polyvalent immunoglobulins (IgG, IgA, and IgM) diluted to 1∶10000 in PBS with 1% BSA for 1 h at room temperature. The membranes were washed again and extravidine-peroxidase was added and membranes were further incubated for 1 h. After the PBS wash, the reaction was developed by adding diaminobenzidine reagent and incubating for 30 min.

The same procedure was performed with the peroxidase-conjugated goat anti-human IgG (Sigma-Aldrich, UK) diluted to 1∶12000 in PBS with 1% BSA.

### Serological expression cloning

#### Isolation of genomic DNA from fecal specimens

Total DNA was isolated directly from the fеcal samples obtained from four FMF patients and two healthy controls (Table 1). The samples were vigorously resuspended in PBS (1∶9) and centrifuged at low speed (700×g) to remove debris. The supernatant was centrifuged at 10,000×g for 10 min to collect bacteria. Bacterial pellets were washed in PBS and TES buffers, inactivated at 80°C and stored lyophilized until DNA isolation. Genomic DNA was isolated with Wizard Genomic DNA purification kit (Promega, UK). The second DNA purification step was done by TE-saturated phenol∶chloroform∶isoamyl alcohol (25∶24∶1) extraction and ethanol precipitation. Finally, DNA was purified by passing through the SPIN Column-1000 (Sigma, UK).

#### Genomic expression library construction

A detailed description of library construction can be found elsewhere [39]. Libraries were generated using the Lambda Zap II predigested /EcoR I/CIAP – treated vector kit (Stratagene, UK). In brief, total genomic DNA was digested with EcoRI restriction enzyme (Promega, UK) and approximately 0.25 µg of fragments ranging from 0.7 kb to 3 kb were ligated to 1.0 µg of the vector. The ligation mix was packed using Gigapack III Gold packaging extract (Stratagene, UK) and the plaque-forming units (PFU) of packaging reaction were titrated following the manufacturer's instructions. Blue or white colony color selection was used to distinguish between non-recombinants and recombinants *E. coli* clones using 5-bromo-4-chloro-3-indolyl-β-D-galactopy-ranoside (X-Gal) on Luria-Bertani (LB) agar plates. Protein expression was induced with 10 mM isopropyl β-thiogalactopyranoside (IPTG).

#### Expression screening

Immunological screening of the libraries was carried out using the pool of corresponding serum samples (Table 1). Immunoreactive proteins were screened on the plates with approximately 6×10^5^ PFU of the unamplified fecal bacteria expression lambda library. Each library was plated on 150-mm agar plates with *E. coli* XL1-Blue MRF' host cells and incubated at 37°C until plaques formed. Immobilon-P transfer membranes (Millipore, UK) pre-wet with 10 mM IPTG were placed on the plates, which were then incubated overnight at 37°C. Filters were removed and washed three times with PBS containing 0.1% Tween 20 (PBST) (Sigma-Aldrich, UK), blocked with 1% BSA in PBST, and washed three times with PBST. The filters then were incubated overnight with *E. coli* lysate-depleted sera of investigated subjects (1∶500 dilution in PBST), washed three times with PBST, and incubated with a anti-human polyvalent immunoglobulins (IgG, IgA, and IgM) peroxidase-conjugated secondary antibody (Sigma-Aldrich, UK) at a dilution of 1∶10000 with PBST for 1 h. Membranes were finally washed and visualized with 3,3′,5,5′-tetramethyl-benzidine (TMB) (Sigma-Aldrich, UK). Reactive plaques were then isolated and subjected to the second and third rounds of purification.

The inserts were recovered by excision using Exassist helper phage according to the manufacturer's instructions (Stratagene, UK) and introduced into *E. coli* SOLR. The resulting recombinant plasmid DNA was sequenced on an automated Beckman sequencer (Beckman, UK) using the M13 forward and reverse primers as well as by primer walking using custom primers. Sequences were assembled as described before for the 16S rRNA gene sequences. Nucleotide and translated amino acid sequences were searched using on-line BLAST (http://www.ncbi.nlm.nih.gov/blast), PFAM (http://www.sanger.ac.uk/Software/Pfam) and PSORT (http://psort.nibb.ac.jp) programs.

### Statistical analyses

Statistical analyses were carried out using the Statsoft Statistica package (www.statsoft.com). Student's t-test for independent samples was applied to determine statistical significance between the mean values of two study groups. P-values below 0.05 were considered statistically significant.

### Accession numbers

Nucleotide sequences generated during this work have been submitted to GenBank under accession numbers EU722733–EU722747.

## Results

### Representatives of gut commensal bacteria

From a total of 120 gut bacterial isolates, 15 were selected for immunological analyses. These bacteria belonged to the *Bacteroides*, *Parabacteroides*, *Enterococcus*, *Escherichia* and *Lactobacillus* genera (Tables 2, 3, 4, 5). Thus the isolates covered a range of typical gut commensal bacteria from the *Bacteroidetes*, *Firmicutes* and *Proteobacteria* phyla.

**Table 2:** Titers of IgG reacting with antigens of commensal bacteria in three investigated groups.

Phylum (genus)	Strain	Antibody titers Mean±SD			OD cut-off value*
		Control	FMF patients		
			Remission	Attack	
*Bacteroidetes (Bacteriodes)*	*Bacteroides ovatus*	890.9±433.1 (11)**	2587.5±1670.0†† (8)	2487.5±1750.8†† (4)	0.8
	*Bacteroides* sp.	1072.7±593.8 (11)	1877.8±1386.5 (9)	2616.7±1695.6† (3)	1.0
	*Bacteroides dorei*	2347.2±1108.2 (9)	3355.5±931.3† (9)	3525.0±721.4 (4)	1.7
	*Bacteroides thetaiotaomicron*	1250.0±490.8 (11)	2155.5±1250.9† (9)	2356.2±1213.5† (4)	0.63
	*Bacteroides finegoldii*	1145.5±743.2 (11)	2047.2±1540.6 (9)	2041.7±1739.7 (3)	1.2
	*Bacteroides fragilis*	800.0±273.4 (11)	2177.8±1387.8†† (9)	1306.2±1300.7 (4)	0.9
	*Bacteroides uniformis*	827.3±383.8 (11)	1725.0±1107.0† (9)	1737.5±1132.4† (4)	0.7
*Bacteroidetes (Parabacteriodes)*	*Parabacteroides distasonis*	658.3±161.5 (9)	2180.5±1148.8††† (9)	2383.3±1207.4††† (3)	2.7
	*Parabacteroides merdae*	995.5±363.3 (11)	2508.3±1241.3††† (9)	2393.7±1069.7†† (4)	0.9
*Proteobacteria (Escherichia)*	*Escherichia* sp.	954.5±261.4 (11)	2136.1±1606.8† (9)	1568.7±1628.2 (4)	1.22
	*Escherichia coli*	742.5±324.1 (10)	2358.3±1051.6††† (9)	2656.2±1592.2†† (4)	1.52
*Firmicutes (Lactobacillus)*	*Lactobacillus delbrueckii*	1297.7±1016.8 (11)	1655.5±1281.0 (9)	1862.5±1593.9 (4)	1.4
	*Lactobacillus reuteri*	1675.0±1280.7 (11)	2322.2±1492.6 (9)	2587.5±1637.3 (4)	0.8
*Firmicutes (Enterococcus)*	*Enterococcus hirae*	1034.1±898.7 (11)	2097.2±1391.3† (9)	1862.5±1520.6 (4)	1.2
	*Enterococcus faecium*	975.0±881.1 (11)	2380.5±1386.0† (9)	2075.0±1419.6 (4)	1.22

* ELISA OD cut-off value used to determine the titer of serum.

** The values in parentheses are the numbers of subjects studied.

† p<0.05 as compared to healthy controls.

†† p<0.01 as compared to healthy controls.

††† p<0.001 as compared to healthy controls.

**Table 3:** Titers of IgA reacting with antigens of commensal bacteria in three investigated groups.

Phylum (genus)	Strain	Antibody titers Mean±SD			OD cut-off value*
		Control	FMF patients		
			Remission	Attack	
*Bacteroidetes (Bacteriodes)*	*Bacteroides ovatus*	1290.9±772.8 (11)**	1938.9±1366.1 (9)	1937.5±1340.6 (4)	0.6
	*Bacteroides* sp.	2018.2±1024.0 (11)	2763.9±797.7 (9)	2306.2±1622.3 (4)	2.2
	*Bacteroides dorei*	1720.5±1322.0 (11)	2108.3±1368.1 (9)	2531.2±1357.3 (4)	1.2
	*Bacteroides thetaiotaomicron*	1040.9±550.3 (11)	1911.1±1396.5 (9)	1787.5±1555.8 (4)	0.9
	*Bacteroides fragilis*	2040.9±1114.6 (11)	2563.9±1072.4 (9)	1881.2±1346.5 (4)	0.6
	*Bacteroides uniformis*	1609.1±737.5 (11)	2102.8±1412.9 (9)	2181.2±1339.1 (4)	1.26
*Bacteroidetes (Parabacteriodes)*	*Parabacteroides distasonis*	1106.8±642.2 (11)	2138.9±1405.3† (9)	1318.7±744.2 (4)	0.75
	*Parabacteroides merdae*	984.1±304.4 (11)	1888.9±1302.1† (9)	1506.2±1138.4 (4)	0.9
*Proteobacteria (Escherichia)*	*Escherichia* sp.	1577.3±855.0 (11)	2488.9±1490.4 (9)	1212.5±804.5 (4)	0.8
	*Escherichia coli*	1265.9±609.7 (11)	2002.8±1380.7 (9)	2237.5±1554.6 (4)	0.5
*Firmicutes (Lactobacillus)*	*Lactobacillus delbrueckii*	1668.2±1263.8 (11)	1838.9±1232.6 (9)	1450±144.5 (4)	0.9
	*Lactobacillus reuteri*	1634.1±1215.3 (11)	2180.6±968.1 (9)	1941.7±1784.7 (3)	0.8
*Firmicutes (Enterococcus)*	*Enterococcus hirae*	1520.5±857.2 (11)	1961.1±1212.1 (9)	1768.7±1188.0 (4)	0.5

* ELISA OD cut-off value used to determine the titer of serum.

** The values in parentheses are the numbers of subjects studied.

† p<0.05 as compared to healthy controls.

**Table 4:** Effect of colchicine therapy on IgG titers.

Phylum (genus)	Strain	Antibody titers Mean±SD			OD cut-off value*
		Control	FMF patients		
			Colchicine treated	Colchicine untreated	
*Bacteroidetes (Bacteriodes)*	*Bacteroides ovatus*	890.9±433.1 (11)**	1981.2±1681.2† (8)	3700.0±600.0††† (4)	0.8
	*Bacteroides* sp.	1072.7±593.8 (11)	2025.0±1465.1 (9)	2175.0±1602.1 (3)	1.0
	*Bacteroides dorei*	2347.2±1108.2 (9)	3222.2±961.1 (9)	3825.0±247.5† (4)	1.7
	*Bacteroides thetaiotaomicron*	1250.0±490.8 (11)	1991.7±1227.1 (9)	2725.0±1081.9†† (4)	0.63
	*Bacteroides finegoldii*	1145.5±743.2 (11)	1888.9±1606.3 (9)	2516.7±1325.1† (3)	1.2
	*Bacteroides fragilis*	800.0±273.4 (11)	1913.9±1518.0† (9)	1900.0±1176.3†† (4)	0.9
	*Bacteroides uniformis*	827.3±383.8 (11)	1400.0±906.3 (9)	2468.7±1144.8††† (4)	0.7
*Bacteroidetes (Parabacteriodes)*	*Parabacteroides distasonis*	658.3±161.5 (9)	2461.1±1201.4††† (9)	1541.7±240.2††† (3)	2.7
	*Parabacteroides merdae*	995.5±363.3 (11)	2169.4±1171.3†† (9)	3156.2±841.2††† (4)	0.9
*Proteobacteria (Escherichia)*	*Escherichia* sp.	954.5±261.4 (11)	1736.1±1561.9 (9)	2468.7±1682.9†† (4)	1.22
	*Escherichia coli*	742.5±324.1 (10)	2602.8±1326.1††† (9)	2106.2±805.8††† (4)	1.52
*Firmicutes (Lactobacillus)*	*Lactobacillus delbrueckii*	1297.7±1016.8 (11)	1500.0±1330.4 (9)	2212.5±1329.5 (4)	1.4
	*Lactobacillus reuteri*	1675.0±1280.7 (11)	2005.6±1393.7 (9)	3300.0±1400.0† (4)	0.8
*Firmicutes (Enterococcus)*	*Enterococcus hirae*	1034.1±898.7 (11)	1961.1±1469.1 (9)	2168.7±1315.5 (4)	1.2
	*Enterococcus faecium*	975.0±881.1 (11)	2005.6±1378.6 (9)	2918.7±1177.6†† (4)	1.22

* ELISA OD cut-off value used to determine the titer of serum.

** The values in parentheses are the numbers of subjects studied.

† p<0.05 as compared to healthy controls.

†† p<0.01 as compared to healthy controls.

††† p<0.001 as compared to healthy controls.

**Table 5:** Effect of colchicine therapy on IgA titers.

Phylum (genus)	Strain	Antibody titers Mean±SD			OD cut-off value*
		Control	FMF patients		
			Colchicine treated	Colchicine untreated	
*Bacteroidetes (Bacteriodes)*	*Bacteroides ovatus*	1290.9±772.8 (11)**	1316.7±938.4## (9)	3337.5±809.7††† ## (4)	0.6
	*Bacteroides* sp.	2018.2±1024.0 (11)	2233.3±1046.2# (9)	3500.0±349.4† # (4)	2.2
	*Bacteroides dorei*	1720.5±1322.0 (11)	1886.1±1141.4 (9)	3031.2±1521.1 (4)	1.2
	*Bacteroides thetaiotaomicron*	1040.9±550.3 (11)	1266.7±1047.2## (9)	3237.5±1060.9††† ## (4)	0.9
	*Bacteroides fragilis*	2040.9±1114.6 (11)	1825.0±922.2## (9)	3543.7±561.4† ## (4)	0.6
	*Bacteroides uniformis*	1609.1±737.5 (11)	1744.4±981.4 (9)	2987.5±1768.4† (4)	1.26
*Bacteroidetes (Parabacteriodes)*	*Parabacteroides distasonis*	1106.8±642.2 (11)	1352.8±857.3# (9)	3087.5±1305.3†† # (4)	0.75
	*Parabacteroides merdae*	984.1±304.4 (11)	1250.0±823.5# (9)	2943.7±1223.1††† # (4)	0.9
*Proteobacteria (Escherichia)*	*Escherichia* sp.	1577.3±855.0 (11)	1494.4±1118.3# (9)	3450.0±1100.0†† # (4)	0.8
	*Escherichia coli*	1265.9±609.7 (11)	1405.6±1047.2## (9)	3581.2±507.2††† ## (4)	0.5
*Firmicutes (Lactobacillus)*	*Lactobacillus delbrueckii*	1668.2±1263.8 (11)	1280.6±892.7# (9)	2706.2±1224.5# (4)	0.9
	*Lactobacillus reuteri*	1634.1±1215.3 (11)	2027.8±1276.7 (9)	2400.0±567.9 (3)	0.8
*Firmicutes (Enterococcus)*	*Enterococcus hirae*	1520.5±857.2 (11)	1463.9±823.5# (9)	2887.5±1297.7† # (4)	0.5

* ELISA OD cut-off value used to determine the titer of serum.

** The values in parentheses are the numbers of subjects studied.

† p<0.05 as compared to healthy controls.

†† p<0.01 as compared to healthy controls.

††† p<0.001 as compared to healthy controls.

# p<0.05 colchicine treated vs. colchicine untreated.

## p<0.01 colchicine treated vs. colchicine untreated.

### p<0.001 colchicine treated vs. colchicine untreated.

### Level of total systemic antibodies

Irrespectively of the disease state, the titer of polyvalent antibodies (IgG, IgA, and IgM) in the serum of FMF patients exceeded that of the healthy subjects by 42%. Among them, the total IgG titer in FMF was elevated by 35% compared to control. Both changes were statistically not significant (data not shown).

### Commensal bacteria-specific antibodies

The level of IgG and IgA towards the commensal bacterial antigens was evaluated by the reactivity of the sera of FMF patients and healthy controls with the corresponding bacterial lysates. For this, total lysates of pure bacterial cultures were coated onto a 96-well plate and probed with the corresponding sera as described in Materials and Methods.

In respect to the disease stage (attack vs. remission) there was little difference in specific IgG or IgA titers directed against the antigens of all commensal bacteria investigated. The differences included the increase of the IgG titer for *Bacteroides* sp. strain and the decrease of it for *B. fragilis* and *Escherichia* sp. strain in attack compared to remission (Table 2). The IgA titers towards the two strains of *Parabacteroides* were lower in attack than in remission (Table 3). The highest difference in specific IgG titers was obtained in the case of *Escherichia* and *Parabacteroides* antigens, with respectively 2.2 to 3.2-fold and 2.5 to 3.3-fold increase of IgG in the sera of FMF patients in comparison with control. Very similar results were obtained using the antigens of strains belonging to the *Bacteroides* genus, where the level of IgG against these bacteria was approximately from 1.4 to 2.9-fold higher in FMF patients than in control (Table 2). Measurement of IgG titers against *Enterococcus* bacteria revealed significant elevation of specific antibodies in FMF subjects, namely in *E. faecium* and *E. hirae*. However, there was no difference in *Lactobacillus*-specific systemic IgG levels in healthy and diseased subjects (Table 2). It is also noteworthy that in healthy subjects the variation of specific titers was quite low, while in the diseased subjects very broad fluctuations of the corresponding parameters were observed.

In contrast to IgG, there was essentially no difference in the level of bacteria-specific IgA in the blood sera of FMF patients and healthy subjects (Table 3). Only the titers of IgA directed against the strains of *Parabacteroides* were elevated in the sera of FMF patients compared to control (p<0.05).

In respect to colchicine treatment, no significant changes in IgG titers between the treated and untreated patients were observed (Table 4). On the other hand, systemic IgA titers against the antigens of commensal bacteria were found to be significantly higher in the sera of colchicine-free FMF patients in comparison with the cohort undergoing the therapy, except *B. uniformis*, *B. dorei* and *L. reuteri* (Table 5). In the case of the last two bacteria, though, there was no statistically significant difference with the healthy cohort either. The level of systemic IgA directed against commensal bacteria was very similar between the colchicine-treated FMF patients and control, suggesting the normalization of this parameter following the colchicine therapy.

### Western blot

**Figure 1:**
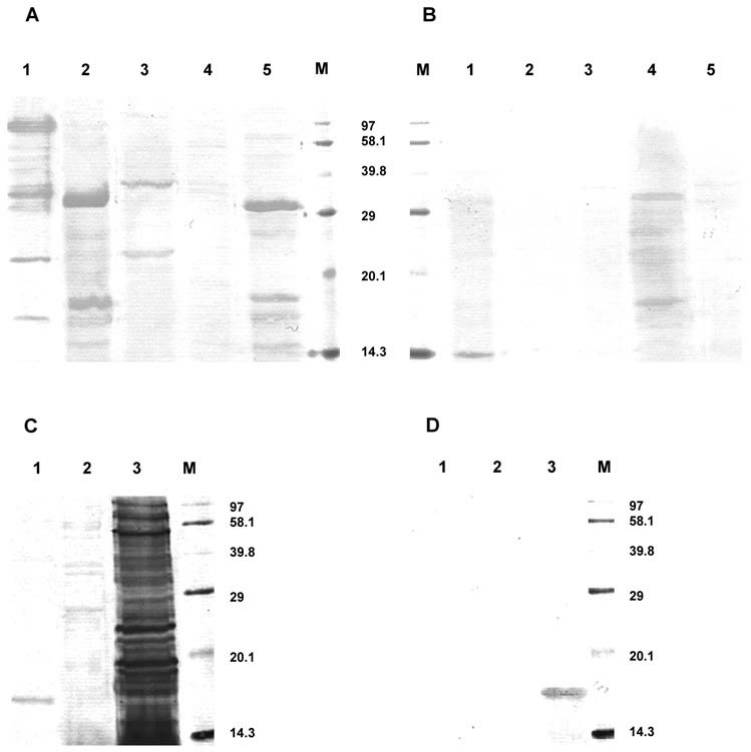
Western blot of bacterial lysates probed with the corresponding sera. A: bacterial lysates probed with the serum of FMF190(A); B – with C110; C – with FMF87(R); and D – with C111. In A and B: line 1 – *B. ovatus*, line 2 – *P. distasonis*, line 3 – *Bacteroides* sp., line 4 – *L. reuteri*, line 5 – *E. coli*, and line M – biotinylated molecular weight standard mixture SDS-6B (Sigma-Aldrich, UK). In C and D: line 1 – *E. hirae*, line 2 – *L. reuteri*, line 3 – *B. ovatus*, and line M – biotinylated molecular weight standard mixture SDS-6B (Sigma-Aldrich, UK).

Bacterial lysates were run on SDS-PAGE, transferred to a membrane and probed with the sera of FMF patients and healthy control subjects, the representative examples are shown in Fig. 1. Since the protein concentration applied to each lane was normalized, the presence of more intense bands on the gels probed with the sera of FMF patients in comparison with control confirms the earlier ELISA findings, that is the blood sera of diseased subjects contains higher concentrations of bacteria-specific antibodies. The Western blot analysis also demonstrated that there are no isolated major bacterial antigens against which the host mounts humoral response. The responses were rather non-specific and included multiple antigens of different commensal bacteria. The most intense responses were seen against the antigens of *B. ovatus*, *P. distasonis*, *E. coli*, and *Bacteroides* sp. but the reactivity against the antigens of *Lactobacillus* and *Enterococcus* was less pronounced (Fig. 1A and 1C), thus confirming our earlier ELISA findings. The response against the *B. ovatus* lysate showed very strong reactivity against numerous antigens, with multiple epitopes, while in the case of *P. distasonis*, *E. coli* and *Bacteroides* sp. the antibodies reacted with fewer major antigens. Interestingly, in some cases the blood sera of healthy control subjects reacted with the same bacterial antigens, although the reactivity was generally very low (Fig. 1B and 1D).

### Serological expression cloning

With the aim of identifying the antigens of commensal gut bacteria that elicit systemic response in FMF we adopted a serological expression cloning approach. For this, we constructed six metagenomic lambda expression libraries from feces of four FMF patients and two healthy subjects and screened them with the corresponding host sera (see Materials and Methods). The libraries from healthy subjects produced no positive clones and all 35 clones isolated were from the libraries of FMF patients (six clones from FMF107, seven clones from FMF155, nine clones from FMF156, and 13 clones from FMF87) (supplementary material, Table S1). The majority of ORFs in these clones had the closest matches with proteins from intestinal *Bacteroides* and *Parabacteroides* species. Most of the ORF-encoded proteins were isolated on a single occasion thus confirming the results of Western blot analysis suggesting that the immune response is directed against a broad range of gut commensal bacterial antigens. The most notable exception was an ORF sharing 28–33% identity with the hypothetical cell surface protein precursor from *Lactobacillus sakei*, which was isolated in eight independent occasions but from the same patient, FMF87 (in clones 18, 20, 21, 28, 30, 31, 36, and 38). Since the libraries were not amplified before the screening, this suggests either the overrepresentation of this gene in the corresponding gut metagenome or possibly an enhanced translocation of this antigen through the gut epithelial barrier. The metagenomic DNA fragment encoding an ORF with 71% similarity to the hypothetical protein PARMER_04074 from *Parabacteroides merdae* was encountered in four occasions, also only in a single patient, FMF155. Clones 53 and 66 from patient FMF156 both contained the identical insert encoding a protein with 82% of similarity to chaperonin GroEL from *Bacteroides vulgatus*. Two other clones from the same patient, 64 and 65, were also identical and encoded a peptide with 54% similarity to the hypothetical protein from *Bacteroides fragilis*. The remaining 20 clones encoded ORFs that were encountered only once during the analysis. One of the ORFs, ORF1 from clone 55 came with an unexpectedly high similarity to the unnamed protein product of *Homo sapiens* (Table S1). More careful inspection with human genome databases, however, failed to confirm that the protein is indeed of the human origin, thus the GenBank entry BAC86097 most likely is an artifact of cloning. Domain structure analysis of translated ORF1 from clone 55 suggested that it is a putative transmembrane CorC/HlyC family transporter protein, with highest similarity to the protein from *Bacteroides coprocola*.

## Discussion

In this work, we demonstrated the elevated level of systemic antibody response towards the antigens of common gut commensal bacteria in autoinflammatory condition. Under the normal circumstances, it is unusual for the systemic adaptive immunity to be primed against the intestinal microbiota and we detected no such responses in healthy subjects. The mechanisms that protect the host from bacterial translocation in the gut include passive physical barriers such the mucus layer that protects the epithelium and traps the bacteria, as well as a number of more pro-active cellular and humoral mechanisms. Defects in any of these defense systems result in severe abnormalities and disease. For example, Muc2-deficient mice spontaneously develop colitis [40] while Ig-deficient mice show hyperplasia of intestinal lymphoid follicles and the overgrowth of a segmented filamentous bacterium [41]. In humans, mutations in NOD2/CARD15 are associated with the decrease in alpha-defensin expression [42], which may predispose an individual to CD. In CD, bacterial translocation, with subsequent generation of strong systemic response is paramount and involves a generalized increase of IgG against commensal intestinal microbiota [22].

In our work, to establish whether an autoinflammatory condition such as FMF also generates a substantial systemic response against the commensal gut microbiota, we initially isolated and identified a number of intestinal bacteria from three FMF patients and a healthy control subject. The rich media and anaerobic conditions used for isolation allowed us to isolate the predominant intestinal bacteria while selective media was used to represent a broader selection of bacterial diversity. According to sequence analysis of the 16S rRNA genes, the most abundant bacteria isolated by culturing appeared to be the species of *Bacteroides*, which coincides with gut molecular diversity analysis [43]. Very few representatives of the *Firmicutes* were isolated, mostly limited to enterococci and lactobacilli. The selected 15 isolates represented the three main phyla (*Bacteroidetes*, *Firmicutes*, and *Proteobacteria*) and five genera (*Bacteroides*, *Parabacteroides*, *Escherichia*, *Enteroccocus*, and *Lactobacillus*) thus covering a broad range diversity to screen for possible antigens originating from gut commensal bacteria.

The cell-free lysates of these bacteria were probed with the blood sera to establish whether the adaptive immune system of FMF patients and healthy control subjects is different in terms of specific antibody titers directed against the gut microbiota. In FMF patients, we observed exaggerated systemic IgG response primed against the harmless intestinal bacteria; the highest titers were against the species belonging to the *Parabacteroides*, *Bacteroides*, *Escherichia* and *Enterococcus* genera. At the same time, the reactivity of systemic IgA antibodies against the epitopes of selected bacteria was not different between FMF patients and control. Need to say, however, that the majority IgA synthesis is confined to lamina propria, where it is produced in quantities that exceeds the sum of all other immunoglobulin isotypes combined [10]. In contrast to secretory IgA at mucosal surfaces, serum IgA, however, is a potent trigger of (pro)-inflammatory activity upon binding to the myeloid IgA receptor, FcαR [44]. We did not detect any substantial increase of systemic IgA levels in FMF patients in comparison with control subjects; in this regard, mucosal IgA seems an attractive target to study, especially in the light of its role in controlling the composition of gut microbiota [41]. We also assessed the influence of colchicine treatment of FMF patients on systemic immunoglobulin isotypes and found its “normalizing” effect on the level of serum IgA that is directed against the antigens of commensal gut bacteria. Historically, the antimitotic drug colchicine has been, and still is, extensively used for the management of consequences of gout and pseudogout, that is, of inflammation attacks [45], which are caused by uric acid crystals that activate the NALP3 inflammasome [32]. In FMF, as we hypothesized before, the lamina propria neutrophils that carry the mutated version of pyrin may have the heightened sensitivity of the NALP3 inflammasome to the bacterial antigens continuously escaping from the lumen and thus the inflammasome may be easily activated, even under the antigen load, which is well tolerated in the norm. Colchicine is also used for the management of Behcet's disease and cirrhosis so is difficult to propose the unifying mechanistic explanation for the positive effect of colchicine for the diseases with such different ethiology. Colchicine may interfere with microtubule formation, thereby affecting mitosis and other microtubule-dependent functions such as diapedesis [46]. As a consequence of the reduced mobility, the infiltration of leukocytes to the affected sites may be impeded, thus reducing the extent of inflammation and tissue damage. In the case of FMF, its positive effect can also be explained by the reduction in bacterial translocation through the gut epithelial barrier as it has been demonstrated for T84 epithelial cells under the metabolic stress [47]. Reduced translocation of bacteria and bacterial ligands due to colchicine therapy may halt the initiation of inflammation by the pyrin-deficient hypersensitive NALP3 inflammasome present in neutrophils, monocytes and dendritic cells circulating in the lamina propria of FMF patients.

Western blot results essentially confirmed ELISA data on the elevated adaptive immunity in FMF patients that is directed against commensal bacteria. The IgG response against *E. coli*, *P. distasonis* and all *Bacteroides* strains, especially *B. ovatus*, was much higher in FMF patients than in controls. More importantly, this analysis added another piece in understanding the nature of adaptive immunity in FMF such as the absence of unique or major bacterial antigen(s) responsible for priming the adaptive immunity. The serum antibodies reacted with a wide range of proteins from different bacteria including *B. ovatus*, *P. distasonis*, *E. coli* and others. Therefore the systemic response in FMF is not directed against a specific epitope or bacterium thus excluding the possibility of the involvement of a specific pathogen in this disease.

With the aim of identifying the nature of these bacterial antigens, we performed serological expression screening of the gut metagenomes. The libraries were not amplified prior the screening to avoid a potential selection of clones. The majority of ORFs was unique and, unlike the Crohn's disease [20], [21], no single dominant antigen, which is characteristic for FMF, can be identified. The best matches were with proteins from intestinal *Bacteroides* and *Parabacteroides* species, which is not surprising since these bacteria belong to one of the two dominant bacterial phyla in the intestine, the *Bacteroidetes* and *Firmicutes* [43]. There were very few sequences from the latter phylum, though, exemplified by ORFs with similarity to proteins from the *Lactobacillus*, *Clostridium*, *Dorea* and *Eubacterium*. Interestingly, the antigen with a 28–33% similarity to the hypothetical surface protein precursor from *L. sakei* was cloned from the fecal metagenome of FMF87 patient eight times, which seems contradict ELISA and Western blot data that suggested a generally very low reactivity of sera against the antigens of *Lactobacillus* species. However, the similarity value is fairly low and we cannot exclude that this ORF was cloned from bacteria other than the lactobacilli. Two clones encoded the ORFs, which translations displayed the best matches with proteins of the *Proteobacteria*, in particular, of *E. coli*. Thus the results of serological expression study confirmed our ELISA and Western blot analyses data suggesting that the systemic immune response in autoinflammatory condition such as FMF is directed mostly against the multiple antigens of the *Bacteroidetes* and *Proteobacteria*. Despite the numerical prevalence of the *Firmicutes* in the gut [43], all three analyses demonstrated very low level of antibodies directed against, and antigens cloned from, this group of bacteria. In the other inflammatory condition, CD, it has been suggested that the majority of bacteria implicated in this disease are the *Firmicutes* and the dominant antigen is the bacterial flagellin from them [20]. At the same time, a recent work suggested that the markers such as antibodies against flagellins are the surrogate markers of a more generalized response against the common intestinal microbiota [22]. In this regard, despite the different genetics and ethiology, both inflammatory conditions, CD and FMF, share the same feature as a generalized adaptive response directed towards gut commensal bacteria.

Under the normal circumstances, adaptive immunity in the gut is confined and limited mostly to mucosal secretory IgA producing cells, including the cells that undergo isotype switching at the mucosal surfaces. Initially, the luminal antigens are sampled by the Peyer's patch M cells and DC, then the antigens are processed and presented to immunocompetent T-cells. The circuit includes the maturation of gut antigen-primed B and T cells in GALT and MLN and through the peripheral blood back to lamina propria following the homing signals. In healthy individuals, the rate of bacterial translocation, which is defined by bacterial analysis of intestinal serosa and mesenteric lymph nodes, is 5–10% [48]. This seems has no any deleterious consequence for the health. In our healthy subjects the antibody level towards gut commensal bacteria was generally low and we were unable to clone any bacterial antigen from the libraries produced from the fecal samples of two control individuals. Thus in the norm the antigens of gut commensal microbiota are not exposed to systemic circulation. It is not clear, however, how the systemic adaptive immunity becomes primed against these bacteria in FMF patients. As we hypothesized before, because of the mutated version of pyrin in FMF, the NALP3 inflammasome in neutrophils, monocytes and dendritic cells of the lamina propria may be highly sensitive to exo- and endogenous stimuli and, in particular, to bacteria/bacterial ligands translocating from the gut lumen. While these physiologically conventional concentrations of bacteria/bacterial ligands are handled without deleterious consequences by the normal immune system, in a genetically susceptible host with the hypersensitive inflammasome it may trigger the IL-1β cascade. Indeed, the massive influx of polymorphonuclear leukocytes into the affected tissues is one of the clinical symptoms of acute FMF (1). The overproduction of nitric oxide by PMN could be one of the factors compromising the integrity of mucosal barrier, by directly increasing its permeability and bacterial translocation [49], [50]. Also, in our previous work we observed extremely high levels of systemic IL-6 in acute FMF, which was still elevated in remission [6]. This cytokine is essential for the development of gut barrier dysfunction following injury [51]. These and possibly other factors in FMF may contribute to the enhanced translocation of commensal gut bacteria resulting in the increased level of systemic antibodies observed in our work.

## Supporting Information

Table S1 Closest matches of ORFs from serologically expressed clones to database entries. This is a supplementary Table S1, which describes the closest matches of ORFs from serologically expressed clones to database entries.
(0.07 MB DOC)
