# Supplementary material for: Elevated Systemic Antibodies towards Commensal Gut Microbiota in Autoinflammatory Condition
Source: PLoS One. 2008 Sep 9;3(9):e3172. doi: 10.1371/journal.pone.0003172 (PMC2525839; doi:10.1371/journal.pone.0003172)
Supplement: Table S1 — Closest matches of ORFs from serologically expressed clones to database entries. This is a supplementary Table S1, which describes the closest matches of ORFs from serologically expressed clones to database entries. (0.07 MB DOC) [file pone.0003172.s001.doc]

Table S1. Closest matches of ORFs from serologically expressed clones to database entries.

| **Patient** | **Clone** | **ORFs 3’-5’** | **Closest match** | **Similarity %** | **Accession number** |
| --- | --- | --- | --- | --- | --- |
| Fully sequenced clones | | | | | |
| FMF107 | 3 | ORF1 | Hypothetical protein from *Bacteroides vulgatus* | 40 | ABR40681 |
|  |  | ORF2 | Hypothetical protein BACCAC_00942 from *Bacteroides caccae* | 45 | EDM22555 |
|  |  | ORF3 | Lysyl-tRNA synthetase from *Thermosinus carboxydivorans* | 50 | EAX47223 |
| FMF107 | 9 | ORF1 | Putative dipeptidase from *Parabacteroides distasonis* | 45 | ABR42961 |
|  |  | ORF2 | Ribosomal protein S8 from *Carboxydothermus hydrogenoformans* | 75 | ABB14423 |
|  |  | ORF3 | Ribosomal protein L6 from *Thermosinus carboxydivorans* | 65 | EAX47590 |
| FMF87 | 20 (also independently isolated as clone 18, 28, 30, 31, 36, and 38) | ORF1 | Hypothetical cell surface protein precursor from *Lactobacillus sakei* | 33 | CAI55712 |
| FMF87 | 21 | ORF1 | Hypothetical cell surface protein precursor from *Lactobacillus sakei* | 28 | CAI55712 |
|  |  | ORF2 | N-acetyltransferase from *Enterococcus faecium* | 42 | EAN10486 |
|  |  | ORF3 | Conserved hypothetical protein from *Lactobacillus salivarius* | 56 | ABD99962 |
| FMF87 | 40 | ORF1 | Isoleucyl-tRNA synthetase from *Parabacteroides merdae* | 100 | EDN84744 |
| FMF156 | 42 | ORF1 | Putative DNA primase from a mycobacteriophage | 33 | AAC18498 |
|  |  | ORF2 | Hypothetical protein BACSTE_01057 from *Bacteroides stercoris* | 33 | EDS15618 |
| FMF156 | 45 | ORF1 | Uracil-DNA glycosylase from *Bacteroides vulgatus* | 68 | ABR41749 |
|  |  | ORF2 | Hypothetical protein BACOVA_01887 from *Bacteroides ovatus* | 60 | EDO12388 |
| FMF156 | 49 | ORF1 | Conserved hypothetical protein from *Clostridium botulinum* | 86 | EDT79985 |
| FMF156 | 53 (also independently isolated as clone 66) | ORF1 | Chaperonin GroEL from *Bacteroides vulgatus* or  60 kDa chaperonin from *Bacteroides vulgatus* | 82 | AAZ80414 or  ABR38064 |
|  |  | ORF2 | conserved hypothetical protein from Bacteroides vulgatus | 47 | ABR40187 |
| FMF156 | 55 | ORF1 | Unnamed protein product [*Homo sapiens*] or  Putative transmembrane CorC/HlyC family transporter associated protein from *Bacteroides coprocola* | 94  75 | BAC86097  ZP_02924397 |
|  |  | ORF2 | Hypothetical protein BcopD_16341 from *Bacteroides coprocola* | 32 | ZP_02924396 |
| FMF87 | 57 | ORF1 | Hypothetical protein DORLON_02524 from *Dorea longicatena* | 100 | EDM62112 |
| FMF155 | 62 | ORF1 | Betaine aldehyde dehydrogenase from *Escherichia coli* | 100 | AAC73415 |
|  |  | ORF2 | Transcriptional regulator from *Escherichia coli* | 98 | ZP_00924986 |
| Partially sequenced clones | | | | | |
| FMF107 | 1 | ORF1 | Hypothetical protein BACSTE_02315 from *Bacteroides stercoris* | 30 | EDS14628 |
| FMF107 | 2 | ORF1 | Galactokinase from *Bacteroides coprocola* | 86 | ZP_02922013 |
|  |  | ORF2 | Hypothetical protein BACOVA_01719 from *Bacteroides ovatus* | 59 | EDO12577 |
| FMF107 | 8 | ORF1 | Hypothetical protein BACSTE_03679 from *Bacteroides stercoris* | 80 | EDS13498 |
| FMF107 | 10 | ORF1 | Glycosyl transferase from Candidatus *Chloracidobacterium thermophilum* | 50 | ABV27314 |
| FMF87 | 56 | ORF1 | Putative inorganic polyphosphate/ATP-NAD kinase from *Bacteroides*  *vulgatus* | 62 | ABR38188 |
| FMF155 | 63 | ORF1 | Conserved hypothetical protein from *Bacteroides vulgatus* | 30 | ABR39099 |
|  |  | ORF2 | Putative exported protein from *Bacteroides fragilis* | 58 | CAH06048 |
| FMF156 | 64 (also independently isolated as clone 65) | ORF1 | Hypothetical protein from *Bacteroides fragilis* | 54 | BAD49296 |
| FMF155 | 69 | ORF1 | Hypothetical protein EUBSIR_00970 from *Eubacterium siraeum* | 64 | EDS01153 |
| FMF155 | 71 (also independently isolated as clone 58, 69, and 61) | ORF1 | Hypothetical protein PARMER_04074 from *Parabacteroides merdae* | 71 | EDN84623 |
| FMF156 | 54 | ORF1 | Putative transposase from *Bacteroides fragilis* | 95 | AAA22911 |
| FMF87 | 23 | ORF1 | Putative nitrogen utilization substance protein from *Bacteroides*  *coprocola* | 78 | ZP_02924086 |
| FMF87 | 29 | ORF1 | Alpha amylase from *Stigmatella*  *aurantiaca* | 60 | EAU63390 |
